# Hot Deformation Behavior of Cu–Sn–La Polycrystalline Alloy Prepared by Upcasting

**DOI:** 10.3390/ma13173739

**Published:** 2020-08-24

**Authors:** Siming Hua, Pingze Zhang, Zili Liu, Lin Yang

**Affiliations:** 1 College of Materials Science and Technology, Nanjing University of Aeronautics and Astronautics, Nanjing 210000, China; huasiming1987@126.com (S.H.); liuzili@nuaa.edu.cn (Z.L.); 2 China Railway Construction Electrification Bureau Group Kangyuan New Material Co., Ltd., Jinjiang 214500, China; yanglin941106@163.com

**Keywords:** Cu–Sn–La alloy, stress–strain, dynamic recrystallization, hot deformation, constitutive equation

## Abstract

In this study, the hot deformation of a Cu–0.55Sn–0.08La (wt.%) alloy was studied using a Gleeble-3180 testing machine at deformation temperatures of 400–700 °C and various strain rates. The stress–strain curve showed that the hot deformation behavior of the Cu–0.55Sn–0.08La (wt.%) alloy was significantly affected by work hardening, dynamic recovery, and dynamic recrystallization. The activation energy Q was 261.649 kJ·mol^−1^ and hot compression constitutive equation was determined as $\;\dot{\varepsilon }={\left[\sin h\left(0.00651\sigma \right)\right]}^{10.2378}\cdot \exp\left(33.6656-\frac{261.649}{\mathrm{RT}}\right).$ The microstructural evolution of the alloy during deformation at 400 °C revealed the presence of both slip and shear bands in the grains. At 700 °C, dynamic recrystallization grains were observed, but recrystallization was incomplete. In summary, these results provide the theoretical basis for the continuous extrusion process of alloys with promising application prospects in the future.

## 1. Introduction

Copper alloys are structural and functional materials with excellent electrical and mechanical properties. These features make them suitable for applications in designing the frame of large-scale integrated circuits, contact wires of electrified railways [1], lining of molds, and conductors for high pulse magnetic fields and traction motor rotors [2], among others.

Many studies have so far been published on the thermal deformation behavior of copper alloys including the Cu–Fe alloy [3,4], Cu–Ni–Si alloy [5,6], Cu–Ag alloy [7], and Cu–Al_2_O_3_ composites [8], Cu–Cr–Zr alloy [9,10], Cu–Cr–Zr–Ce, Nd, Y alloy [11,12,13], Cu–Mg alloy [14,15], and Cu–Al alloy [16,17]. However, only a few studies have been published on Cu–Sn alloys used in the contact wires of electrified railways. On the other hand, the continuous extrusion process is advantageous in terms of low energy consumption and high yield, thereby widely used in the production of contact wires for electrified railways. However, contact wires based on copper alloys still suffer from limitations such as high deformation temperatures, large deformation resistances, and complex thermal deformation behaviors. As a result, the optimization process of continuous extrusion remains extremely complex, and studies dealing with the hot deformation behavior of Cu–0.55Sn alloy should help optimize the deformation behavior of Cu–Sn alloys via the continuous extrusion process. Furthermore, the addition of small amounts of rare earth elements into copper alloys could purify the matrix and grain boundary, improve the conductivity as well as improve the softening temperature and strength of the alloy [18]. For instance, the performance of Cu–0.55Sn alloys could be improved by adding 0.08% La.

In this study, the hot deformed behavior of a Cu–0.55Sn–0.08La alloy was studied in an effort to provide the theoretical basis for optimizing the continuous extrusion process. The results indicated that the hot deformed behavior of Cu–0.55Sn–0.08La (wt.%) alloy was significantly affected by work hardening, dynamic recovery, and dynamic recrystallization. The activation energy Q and constitutive equation of hot deformation were determined by examining the relation among hot compression flow stress and strain, strain rate, and deformation temperature of Cu–0.55Sn–0.08La alloy.

## 2. Materials and Methods

First, electrolytic copper (purity 99.99%), Sn (purity 99.95%), and pure block La (purity 99.5%) were together melted in a power frequency induction furnace. The molten copper liquid was then continuously cast into a Cu–Sn–La alloy rod billet (diameter 20 mm) using an up-casting machine for further use. The mass fractions of alloy elements were determined as 0.55% Sn, 0.08% La, and Cu balance. Next, the continuous casting rod (diameter 20 mm) was cut by Lathe and Wire Electrical Discharge Machining (WEDM, Taizhou, China) into samples with a size of Ф 8 mm × 12 mm. The specification of the properties of Cu–Sn–La alloy are shown in Table 1.

**Figure 1:**
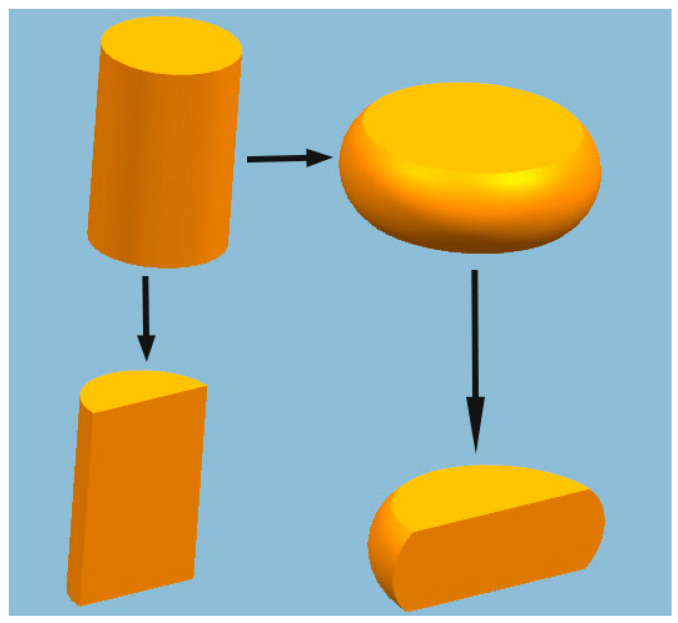
Schematic illustration of hot compression and metallographic samples.

The isothermal compression tests were conducted using a Gleeble-3180 simulator at deformation temperatures ranging from 400 to 700 °C (400, 500, 600, and 700 °C) and strain rates from 0.01 to 10 s^−1^ (0.01, 0.05, 0.1, 1, and 10 s^−1^). Under each condition, compression tests were carried out once. Before isothermal compression, all specimens were heated to the deformation temperature at a heating rate of 5 K·s^–1^ and maintained at the deformation temperature for 180 s. The specimen was then compressed to 40% of the original height. Before testing, the two ends of each specimen were lubricated to prevent uneven deformation during hot compression deformation. After compression testing, the specimens were immediately quenched in water to maintain the state of the deformed tissue. The un-deformed and deformed specimens were then sectioned parallel to the compression axis (Figure 1). The specimens were mechanically polished and then etched in a solution containing FeCl_3_ (3 g), HCl (2 mL), and C₂H₆O (96 mL). The microstructures were examined by optical microscopy (OM, LEICA DM2500M, Wetzlar, Germany) and scanning electron microscope (SEM, EDAX-TSL, Burgen, KS, USA).

## 3. Results and Discussion

### 3.1. Stress–Strain Behaviors

**Figure 2:**
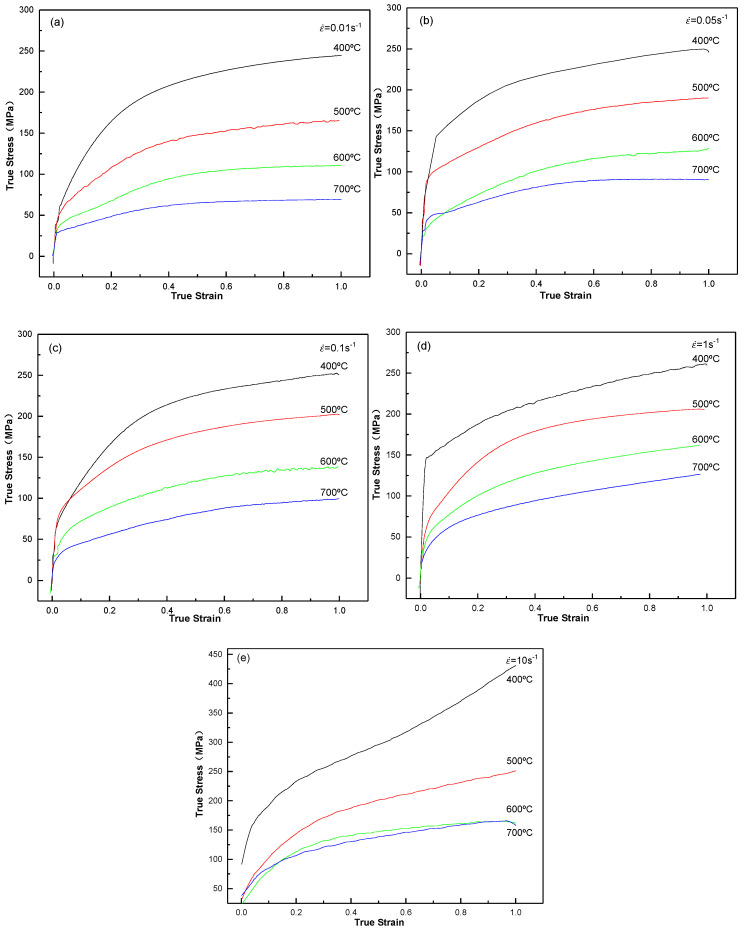
True stress–true strain behaviors of the Cu–0.55Sn–0.08La alloy subjected to various strain rates at different temperatures: (**a**) $\dot{\varepsilon }$ = 0.01 s^−^^1^, (**b**) $\dot{\varepsilon }$ = 0.05 s^−^^1^, (**c**) $\dot{\varepsilon }$ = 0.1 s^−^^1^, (**d**) $\dot{\varepsilon }$ = 1 s^−1^, and (**e**) $\dot{\varepsilon }$ = 10 s^−1^.

The stress–strain behaviors of the Cu–0.55Sn–0.08La alloy at various strain rates and deformation temperatures are displayed in Figure 2. The mechanical energy of the specimen is converted into heat energy during compression; therefore, the temperature rise of the sample is large at a high strain rate, thus it is necessary to modify the experimental data of the stress–strain curve for a strain rate of 10 s^−1^ with temperature [19], and Figure 2e exhibits the modified curve. At fixed deformation temperature, both the flow stress and peak stress increased with strain rate, indicating the positive strain rate sensitivity of the alloy; at fixed strain rate, both the flow stress and peak stress declined with temperature, suggesting the heat-sensitive nature of the alloy [20].

**Figure 3:**
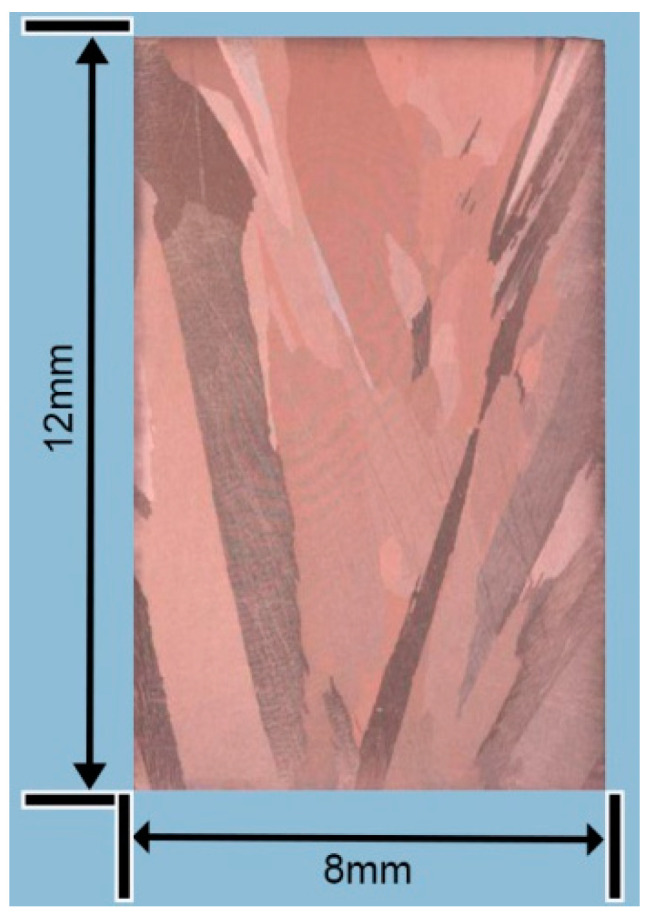
Macrostructure of uncompressed specimen.

The shape of the flow curves exhibited dependence on the initial grain size and steady DRX (dynamic recrystallization) grain size [21]. Figure 3 is initial grain of uncompressed specimen and Figure 7 is the partial recrystallized grain of the compressed deformation alloy. Compared to the initial grain size (1.40 mm) of the test sample was significantly large, the recrystallized grain size (0.06 mm) after dynamic compression. As a result, no peak or only one peak appeared in the stress–strain curve.

Moreover, the shape of each flow curve strongly depended on the solution concentration [22].

The flow stress of the copper alloy was always higher than that of the pure copper under the same temperature and strain rate. The mass fractions of Sn and La in the specimens were determined as 0.55 and 0.08%, respectively. Both the Cu–La intermetallic compounds and Sn solute elements increased the dislocation movement difficulty in the copper matrix, thereby increasing the flow stress of the copper alloy.

At the strain rates of 0.01–1 s^−1^ and 400 °C, the flow stress first increased rapidly with strain and then tended to increase slowly, showing typical work hardening features. At strain rates of 10 s^−1^, the flow stress increased faster than the low strain rate due to the obvious work hardening effect. The final stage of the curve still displayed an upward trend, indicating the dominance of the work hardening. As the strain rate increased, the peak of flow stress increased slowly from 0.01 to 1 s^−1^. For strain rates exceeding the critical value (1 s^−1^), the peak of flow stress increased significantly. When the strain rate increased rapidly, the plastic deformation occurred in a short time, the deformed grains could not recover or recrystallize in time, the work hardening effect was significant, the dislocation density in the alloy increased, and the flow stress peak value increased significantly.

At 500–600 °C, the flow stress first of all increased rapidly with strain and then tended to stabilize without obvious flow stress peaks and sharp softening trend. With the increase of strain rate from 0.01 to 0.05 s^−1^ at 500 °C, the peak value of flow stress increased rapidly and then tended to stabilize from 0.1 to 1 s^−1^. At strain rates exceeding 1 s^−1^, the peak value of the flow stress enhanced significantly. The peak flow stress would enhance significantly, once the strain rate exceeded 1 s^−1^. At 600 °C, from 0.01 to 1 s^−1^, the peak flow stress further increased. As the strain rate reached 1 s^−1^, the peak flow stress hardly increased.

At 700 °C, the flow stress increased rapidly with strain and then tended to stabilize, indicating the important role of dynamic softening in the process. As strain rate incremented, the peak value of the flow stress increased gradually. At 0.01–0.05 s^−1^, the flow stress tended to stabilize as the strain increased. From 0.1 to 10 s^−1^, the flow stress tended to increase slightly with further increase in strain.

Above all, in the range of 400–700 °C, 0.01–10 s^−1^, during the compression experiment, strain hardening and dynamic recovery occurred simultaneously at lower temperature or higher strain rate. With dislocation proliferation, accretion, recombination, and annihilation, the dislocation distribution was observed first to be uneven and then gradually evolved into an independent cellular structure in different dislocation tangled areas [21]. This led to the formation of dislocation cells and reduction in dislocation density. Consequently, the stress–strain curve increased slowly at 400 °C (Figure 2a,b). At high deformation temperatures or low strain rates, the deformation process was accompanied by the formation and growth of recrystallized crystal nuclei, and the softening rate of the alloy appeared greater than or equal to the deformation hardening rate [23]. Thus, the stress–strain curve tended to stabilize (Figure 2a,b at 700 °C). At a deformation hardening rate equivalent to dynamic recovery and dynamic recrystallization rate, the stress–strain curve was stable (Figure 2a,b at 600 °C). The reason for the above-mentioned different laws can be attributed to the competition between dynamic hardening and dynamic softening phenomena.

On the other hand, since the alloy was characterized with low stacking fault energy, its extended dislocations were very wide. Moreover, the dislocations were difficult to extricate from the dislocation network as well as challenging to offset each other through cross slip and climb. At the beginning of deformation, the recovery of the sub-structure was very slow. This led to very high dislocation density in the sub-structure, a very small structure of the sub crystal, and many dislocation tangles in the cell wall.

### 3.2. Microstructure

The macrostructure of an uncompressed specimen is presented in Figure 3. The morphology was generated by different orientations of casting grains, where a single-phase microstructure was etched into different colors. At the edge of the ingot, oblique columnar crystals were formed attributed to the horizontal cooling direction and upward vertical movement. The cooling rate of the ingot center decreased and a few grains with smaller sizes appeared. The average length of the grains on the right side was estimated to be about 3 mm and width was around 0.5 mm. The average length of the grains on the left side was about 10 mm and the width was around 1 mm. Furthermore, the grains on the left and right sides showed obvious boundaries. This was caused by the different cooling rates on both sides of the ingot.

**Figure 4:**
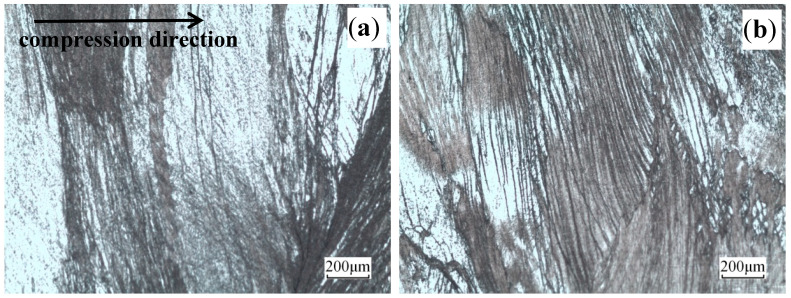
Microstructures of the alloy deformed at 400 °C and different rates: (**a**) $\dot{\varepsilon }$ = 0.01 s^−^^1^ and (**b**) $\dot{\varepsilon }$ = 10 s^−^^1^.

The microstructures of the deformed Cu–Sn–La alloy at 400 °C under different strain rates are illustrated in Figure 4. Due to the large initial grain size, macro coordinated deformation is difficult. The deformation of each grain appeared to be extremely uneven. Moreover, many slip bands and adiabatic shear bands were present in some grains [24], which were terminated at the grain boundary [25,26]. Under compression deformation, the grains rotated to become gradually perpendicular to the compression direction. Compared to Figure 4a, the slip bands in the grains became denser in Figure 4b as strain rate increased.

**Figure 5:**
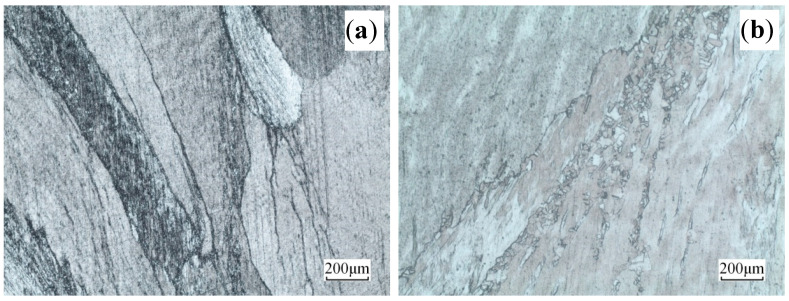
Microstructures of the alloy deformed at 500 °C and different rates: (**a**) $\dot{\varepsilon }$ = 0.01 s^−^^1^ and (**b**) $\dot{\varepsilon }$ = 10 s^−^^1^.

The microstructures of the alloy deformed at 500 °C and different strain rates are presented in Figure 5. At low strain rate ($\;\dot{\varepsilon }$ = 0.01 s^−1^), shear bands still existed and dynamically recrystallized grains appeared in the shear bands (Figure 5a). Thus, the dynamic recrystallization occurred locally with the formation of large numbers of fine dynamic recrystallized grains at the grain boundary. This led to of the formation of large numbers of “necklace structures”. As strain rate increased, at higher strain rate ($\;\dot{\varepsilon }$ = 10 s^−1^), numerous fine recrystallized grains and annealing twins appeared (Figure 5b). In Figure 5b, the wave-like grain boundaries are usually observed under DRX conditions; noteworthy, Figure 5b exhibits that annealing twins were evolved in dynamic grains, although their density was lower than that for statically annealed grains [22].

**Figure 6:**
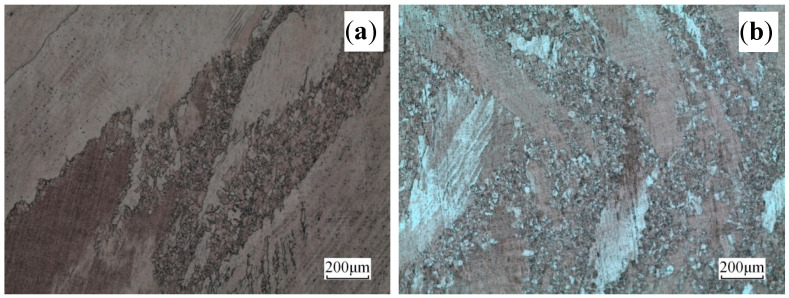
Microstructures of the alloy deformed at 600 °C and different rates: (**a**) $\dot{\varepsilon }$ = 0.01 s^−1^ and (**b**) $\dot{\varepsilon }$ = 10 s^−1^.

The microstructures of the alloy deformed at 600 °C and different strain rates are provided in Figure 6. At low and high strain rates ($\;\dot{\varepsilon }$ = 0.01 s^−^^1^ and $\;\dot{\varepsilon }$ = 10 s^−1^), dynamically recrystallized grains were noted and became obvious as strain rate increased (Figure 6b). Furthermore, the grain boundary preferentially nucleated (Figure 6), and dynamically recrystallized grains gradually expanded and grew around by devouring the surrounding deformed matrix. The latter was due to the grain boundary, which possessed basic conditions of recrystallization nucleation of large-angle interface with high-density defects and superior deformation energy. At this place, recrystallization exhibited priority for nucleation and growth, forming fine and equiaxed recrystallization structures.

**Figure 7:**
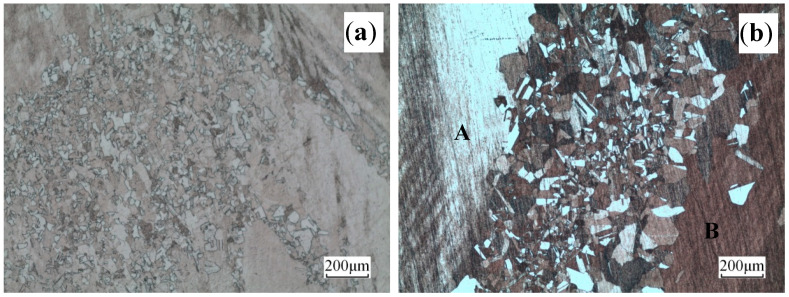
Microstructures of the alloy deformed at 700 °C and different rates: (**a**) $\dot{\varepsilon }$ = 0.05 s^−1^ and (**b**) $\dot{\varepsilon }$ = 10 s^−1^.

The microstructures of the alloy deformed at 700 °C at different strain rates are displayed in Figure 7. Fine recrystallization in the center of Figure 7b was observed, A and B were coarse original grains, and the boundary between the recrystallized grain and original grain appeared to be clear. During dynamic recrystallization, the La rich phase prevented the grain boundary from migrating, thereby reducing the size of the dynamic recrystallization grain.

On the other hand, the recrystallized grain sizes at high strain rates were larger at the same temperature since higher deformation temperatures led to higher thermal activation energy. Furthermore, more complete thermal activation processes led to less storage energy after deformation, thus delaying recrystallization and forming smaller recrystallized grain sizes at low strain rates [27].

**Figure 8:**
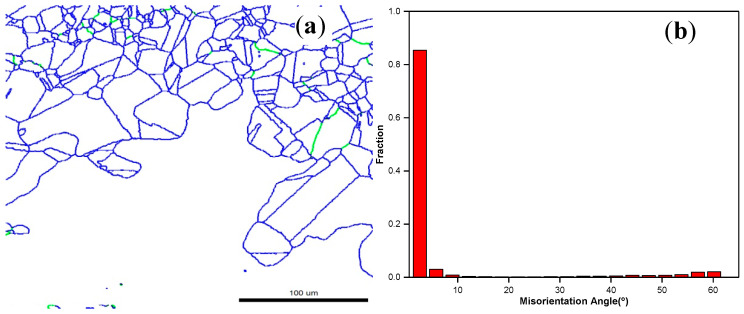
Grain boundary map (**a**) and misorientation distribution diagram (**b**) of the alloy deformed at 700 °C and 10 s^−1^.

Figure 8a show the grain boundary map of the deformed alloy at 700 °C and 10 s^−1^, where the green frame is the recrystallized structures, and the blue frame represents the substructures and deformed structures. Figure 8b shows the misorientation distribution diagram of the deformed alloy at 700 °C and 10 s^−1^, where the fraction of misorientations below 3° was 85%, the fraction of misorientations ranging from 3 to 15° was 4%, and the fraction of misorientations over 15° was 10%. In the microstructure, when the misorientation was less than 3°, the grains were considered as a deformed structure; in the misorientation range (between 3° and 15°), the grains were regarded as substructures; when the misorientation exceeded 15°, the grains were considered as a recrystallized structure [28]. This result explains the shape change of the stress–strain curve (Figure 2)

### 3.3. Constitutive Equations

Under hot processing conditions, constitutive equations are often used to calculate forces during processing at certain setting rates. The modeling of the processing stage must consider the uneven distribution of strain, strain rate, and temperature as well as their variations with time. The model may require several constitutive functions depending on the complexity of the flow curves [29]. The Arrhenius equation can be used to describe the constitutive relation of flow stress behavior during hot deformation. This type of constitutive relation is justified when strain hardening can be ignored. At 400 °C, the strain hardening effect is significant, in particular, in the case of high strain rate (10 s^−1^). At 500–600 ℃, the strain hardening effect is slight. Therefore, the calculation process of the constitutive equation was mainly carried out at 500, 600, and 700 °C.

By the Zener–Holloman parameter (Z), that is, the temperature-compensated strain rate, the relationship between the temperature and the strain rate corresponding to plastic deformation can be analyzed. According to Equation (1) [30], the respective constitutive equation can be expressed as follows:

(1) $$Z=A\cdot {\left[\sin h\left(\alpha \sigma \right)\right]}^{n}=\;\dot{\varepsilon }\cdot \exp\left(Q/\mathrm{RT}\right)$$

In Equation (1), the parameters A, α, and n are constants, which are independent of temperature; the σ represents the true stress in MPa; $\dot{\varepsilon }$ represents the strain rate in s^−1^; the apparent activation energy of deformation is represented by Q in J·mol^−1^; R is equal to 8.314 J·mol^−1^·K^−1^; and T stands for thermodynamic temperature in K.

Equation (2) [31] can adequately express the influence of temperature and strain rate on flow stress, as follows:

(2) $$\;\dot{\varepsilon }=A\cdot {\left[\sin h\left(\alpha \sigma \right)\right]}^{n}\cdot \exp\left(-Q/\mathrm{RT}\right)$$

Notably, according to different stresses, Equation (2) can be transformed into three different forms: when ασ < 0.8, Equation (2) can be transformed into power function Equation (3), when ασ > 1.2, Equation (2) can be transformed into exponential function Equation (4) [32], and the hyperbolic sine function (Equation (2)) is suitable for given any stress.

(3) $$\;\dot{\varepsilon }=A_{1}\cdot \sigma^{n_{1}}\cdot \exp\left(-\frac{Q}{\mathrm{RT}}\right)\;\alpha \sigma <0.8$$

(4) $$\;\dot{\varepsilon }=A_{2}\cdot \exp\left(\beta \sigma \right)\cdot \exp\left(-\frac{Q}{\mathrm{RT}}\right)\;\alpha \sigma >1.2$$

where A_1_ and A_2_ are material constants, and n_1_ and β are related to the strain rate sensitivity index.

Equations (2)–(4) can be written as:

(5) $$\ln\dot{\varepsilon }=\mathrm{nln}\left[\sin h\left(\alpha \sigma \right)\right]+\mathrm{lnA}-\left(\frac{Q}{\mathrm{RT}}\right)$$

(6) $$\ln\dot{\varepsilon }=n_{1}\ln\sigma +{\mathrm{lnA}}_{1}-\left(\frac{Q}{\mathrm{RT}}\right)\;\alpha \sigma <0.8$$

(7) $$n\dot{\varepsilon }=\beta \sigma +{\mathrm{lnA}}_{2}-\left(\frac{Q}{\mathrm{RT}}\right)\;\alpha \sigma >1.2$$

**Figure 9:**
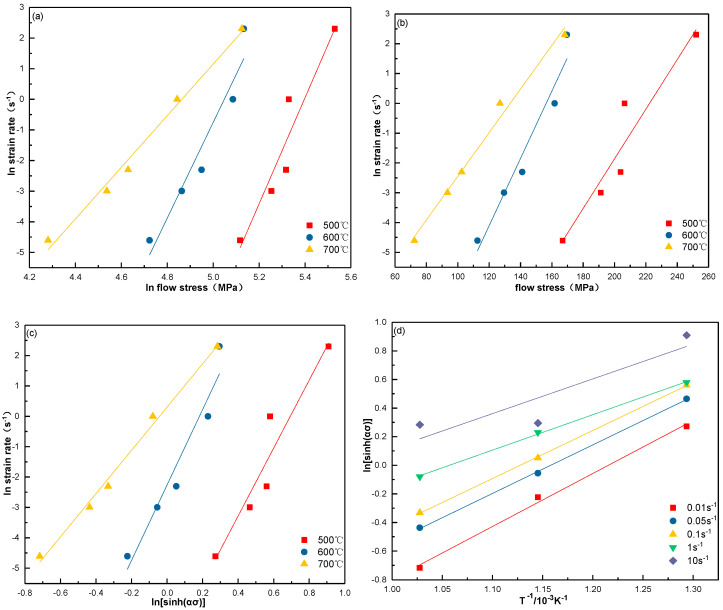
Variations of peak stress (σ) with strain rate ($\dot{\varepsilon }$) and deformation temperature (T) of Cu–0.55 Sn–0.08 La alloy: (**a**) lnσ versus ln$\dot{\varepsilon }$, (**b**) σ versus ln$\dot{\varepsilon }$, (**c**) ln$\dot{\varepsilon }$ versus ln[sinh(ασ)], and (**d**) T−1 versus ln[sinh(ασ)].

The parameters n_1_,  β, and n were calculated by plotting$\;\ln\dot{\varepsilon }$ versus lnσ (Figure 9a), $\ln\dot{\varepsilon }$ versus σ (Figure 9b), and $\ln\dot{\varepsilon }$ versus ln[sinh(ασ)] (Figure 9c), respectively. The material constant n_1_ was estimated by the average of the three slopes of the linear fits of $\ln\dot{\varepsilon }$ versus lnσ with lower peak stress. β was determined by the average of the three slopes of the linear fits of $\ln\dot{\varepsilon }$ versus σ with higher peak stress and α was equal to β divided by n_1_. The slope of the linear fit between $\ln\dot{\varepsilon }$ and ln[sinh(ασ)] is represented by the constant n. Accordingly, the value of n_1_ was estimated to be 13.7935, β was 0.0898, α was 0.00651, and n was 10.2378.

Equation (8) represents the apparent activation energy Q of plastic deformation.

(8) $$\;Q=1000\cdot R\cdot {\left[\frac{\partial \ln\dot{\varepsilon }}{\partial \ln\left[\sinh\left(\alpha \sigma \right)\right]}\right]}_{T}\cdot {\left[\frac{\partial \ln\left[\sinh\left(\alpha \sigma \right)\right]}{\partial \left(\frac{1000}{T}\right)}\right]}_{\;\dot{\varepsilon }}=1000\mathrm{nRK}$$

where K = ${\left[\frac{\partial \ln\left[\sinh\left(\alpha \sigma \right)\right]}{\partial \left(\frac{1000}{T}\right)}\right]}_{\dot{\varepsilon }}$ is the slope of linear fit in Figure 9d.

Accordingly, K was calculated as 3.073796 and Q was 261.649 k·J·mol^−1^. However, the apparent activation energy of the thermal deformation of pure copper with different impurity contents is around 208–245 k·J·mol^−1^ [22,33]. Moreover, higher impurity contents should yield greater activation energies of thermal deformation. The activation energy of the thermal deformation of Cu–0.55Sn–0.08La alloy was estimated to be 261.649 k·J·mol^−1^, indicating that the addition of Sn and La to the copper matrix increased the flow stress of the alloy. This result may be attributed to the interaction between solute atoms Sn and dislocations and grain boundaries, which hindered the dislocation sliding, climbing, and grain boundary migration. These features were unfavorable to the nucleation and growth of recrystallization, thereby limiting the recrystallization process. On the other hand, rare earth La and impurity atoms in liquid copper formed high melting point compounds, which dispersed on the grain boundary. During compression deformation, the dispersed phase was pinned at the grain boundary of the copper alloy, thus hindering the migration of the grain boundary. Moreover, the activation energy of the Cu–0.55Sn–0.08La alloy was found to be higher than that of pure copper.

**Figure 10:**
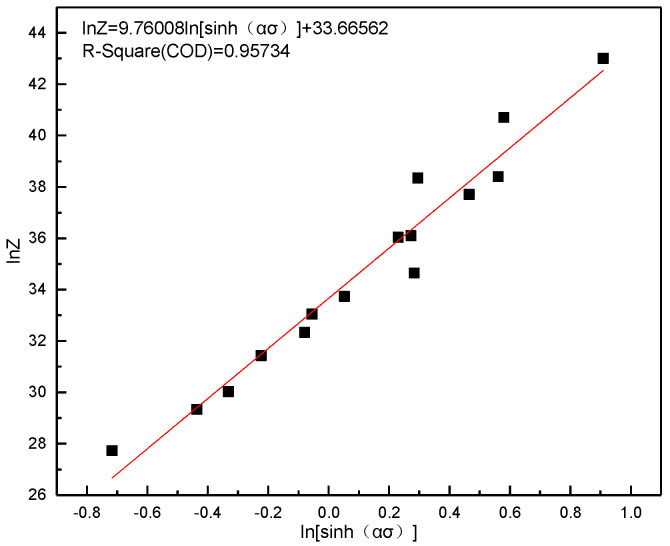
Plot of ln Z versus ln[sinh(ασ)] for evaluating lnA.

In order to calculate n and lnA, ln[sinh(ασ)] was fitted linearly as a function of lnZ and the results are provided in Figure 10. The value of n was estimated to be 9.76008 and lnA was 33.66562.

Based on the above-mentioned analyses, the constitutive equation of Cu–0.55Sn–0.08La at high temperatures was determined as: $\;\dot{\varepsilon }=A\cdot {\left[\sin h\left(\alpha \sigma \right)\right]}^{n}\cdot \exp\left(-\frac{Q}{\mathrm{RT}}\right).$

As a result, Equation (9) can be deduced as:

(9) $$\;\dot{\varepsilon }={\left[\sin h\left(0.00651\sigma \right)\right]}^{10.2378}\cdot \exp\left(33.6656-261.649/\mathrm{RT}\right)$$

## 4. Conclusions

Hot deformation of Cu–0.55Sn–0.08La (wt.%) alloy was successfully studied using a Gleeble-3180 testing machine at deformation temperatures (400–700 °C) and various strain rates. The following conclusions were drawn:

1. The flow stress of the Cu–0.55Sn–0.08La alloy decreased with the deformation temperature and increased with strain rate. At low temperature (400 °C) or high strain rates (1 and 10 s^−1^), the stress–strain curve increased with deformation. At high deformation temperature (700 °C) or low strain rates (0.01 and 0.05 s^−1^), the deformation process was accompanied by the formation and growth of recrystallized nuclei. Furthermore, the softening rate of the alloy was equal to the deformation hardening rate, and the stress–strain curve tended to stabilize.

2. At 500–700 °C and 0.01–10 s^−1^, the relationship between peak flow stress of the Cu–0.55Sn–0.08La alloy and strain rate was determined as: $\;\dot{\varepsilon }=A\cdot {\left[\sin h\left(\alpha \sigma \right)\right]}^{n}\cdot \exp\left(-Q/\mathrm{RT}\right)$ and thermal activation energy Q was 261.649 k·J·mol^−1^. Thus, the constitutive equation can be expressed as: $\;\dot{\varepsilon }={\left[\sin h\left(0.00651\sigma \right)\right]}^{10.2378}\cdot \exp\left(33.6656-\frac{261.649}{\mathrm{RT}}\right)$.

3. The microstructure of Cu–0.55Sn–0.08La alloy showed the presence of slip bands and shear bands in the grains at the deformation temperature of 400 °C. Recrystallization grains were noticed near the shear band at the grain boundary as the deformation temperature increased. At 700 °C, the dynamic recrystallization appeared to be relatively complete with the growth of recrystallization grains.

## Figures and Tables

**Table 1:** Specification of the properties of Cu–Sn–La alloy.

	Tensile Strength/MPa	Rockwell Hardness/HRA	Elongation/%
Cu–0.55Sn–0.08La	223.9	80.2	45.4

## References

[1] Wu P.Y., Xie S.S., Huang G.J. (2006). Materials and Process Technics of Copper Contact Wires for High-Speed Train. Chin. J. Rare Met..

[2] Zhang Y., Xu Q.-Q., Chai Z., Tian B.-H. (2015). High temperature deformation behavior and deformation mechanism of Cu-Cr-Zr-Ag alloy. Chin. J. Nonferrous Met..

[3] Zhang H.-G., Zhang H., Peng D.-S. (2003). Rheologic Stress of C194 Copper Alloy Under Hot Compression Deformation. Natur. Sci. J. Xiangtan Univ..

[4] Yao S., Liu P., Liu X.K. (2013). Hot Plastic Deformation Behavior of KFC Copper Alloy. Shanghai Nonferrous Met..

[5] Liu P., Fan L., Jia S.G., Tian B.H. (2009). Study on dynamic recrystallization behavior and microstructure evolution of Cu-Ni-Si-Cr alloy. J. Univ. Shanghai Sci. Technol..

[6] Zhang Y., Liu P., Tian B.-H., Liu Y., Li R.-Q., Xu Q.-Q. (2013). Hot deformation behavior and processing map of Cu–Ni–Si–P alloy. Trans. of Nonferrous Met. Soc. China.

[7] Wang M.-H., Yang Y.-C., Tu S.-L., Wei K. (2019). A modified constitutive model and hot compression instability behavior of Cu-Ag alloy. Trans. Nonferrous Met. Soc. China.

[8] Shen K., Wang M.P., Guo M., Li S. (2009). Study on High Temperature Deformation Characteristics of Cu-0.23%Al_2_O_3_ Dispersion-strengthened Copper Alloy. Acta Metall. Sin..

[9] Zhang Y., Chai Z., Xu Q.-Q. (2015). Hot deformation behavior and processing maps of Cu-0.8Cr-0.3Zr alloy. Trans. Mater. Heat Treat..

[10] Chen Y., Dang S.E., Ma Y.X., Huo X.B. (2020). High temperature thermal deformation behavior of Cu-Cr-Zr alloy. Forg. Stamp. Technol..

[11] Tian K., Tian B.-H., Liu Y. (2018). Hot Deformation Behavior of Cu-1.0%Zr-0.15%Y Alloy with High Zr Content. Rare Metal. Mat. Eng..

[12] Xu Q.-Q., Zhang Y., Chai Z., Tian B.-H. (2015). High temperature deformation behavior and microstructure evolution of Cu-Cr-Zr-Nd alloy. Chin. J. Nonferrous Met..

[13] Li R.Q., Tian B.-H., Zhang Y. (2013). Hot deformation behavior of Cu-Cr-Zr-Ce alloy at elevated temperature. J. Funct. Mater..

[14] Dai J.Y., Mu S.G., Wang Y.R. (2011). Study on Hot Deformation Behavior and Processing Maps of Cu-0.35Cr-0.15Zr Alloy. Adv. Mater. Res..

[15] Sun G.Q., Liu Y., Tian B.-H. (2018). Hot deformation behavior and mechanism of Cu-0.8Mg alloy. Trans. Mater. Heat Treat..

[16] Gronostajski Z. (2002). The deformation processing map for control of microstructure in CuAl9.2Fe_3_ aluminium bronze. J. Mater. Process. Technol..

[17] Yang C.X., Chen Z.P., Li H.Q. (2010). Flow Stress Feature of Aluminum Bronze Alloy under Hot Deformation. Rare Metal Mat. Eng..

[18] Li Y., Liu Y., Tian B.-H. (2016). Hot Compression Deformation Behavior and Processing Map of Cu-0.4Zr-0.15Ce Alloy. J. Chin. Soc. Rare Earths.

[19] Chen S.C., He S.X., Wu B.J. (1993). The Collating and Correcting of Data in Hot Compress Ing Test For Gleeble Material Hot Modeling Test Machin E. J. Tangshan Instit. Tech..

[20] Li R.-Q., Tian B.-H., Zhang Y., Xu Q.-Q. (2014). Effects of Y addition on hot deformation behavior of Cu-Cr-Zr alloy at elevated temperature. Trans. Mater. Heat Treat..

[21] Sakai T. (1995). Dynamic recrystallization microstructures under hot working conditions. J. Mater. Process. Technol..

[22] Gao W., Belyakov A., Miura H., Sakai T. (1999). Dynamic recrystallization of copper polycrystals with different purities. Mater. Sci. Eng. A.

[23] Srinivasan N., Prasad Y., Rao P.R. (2008). Hot deformation behaviour of Mg–3Al alloy—A study using processing map. Mater. Sci. Eng. A.

[24] Li J.W., Li M.Y., Guo X.L. (2005). Formation of Adiabatic Shear Bands in The Fatigued Copper Single Crystal Deformed at High Strain Rate. Acta Metall. Sin..

[25] Li J.W., Lin D.C. (2006). Metallographic Atlas of Metal Materials.

[26] Humphreys F., Hatherly M. (2004). Recrystallization and Related Annealing Phenomena.

[27] Yu Y.N., Yang P., Qiang W.J. (2006). Fundamentals of Materials Science.

[28] Zhang Y., Jiang S.-Y., Wang S., Sun N., Hu L. (2017). Influence of partial static recrystallization on microstructures and mechanical properties of NiTiFe shape memory alloy subjected to severe plastic deformation. Mater. Res. Bull..

[29] McQueen H., Ryan N. (2002). Constitutive analysis in hot working. Mater. Sci. Eng. A.

[30] Madan P., Ari S., Mahesh C. (2020). Constitutive modelling of hot deformation behaviour of a CoCrFeMnNi high-entropy alloy. Sci. Technol. Adv. Mater..

[31] Zhang L., Li Z., Lei Q., Qiu W., Luo H. (2011). Hot deformation behavior of Cu–8.0Ni–1.8Si–0.15Mg alloy. Mater. Sci. Eng. A.

[32] Ryan N., McQueen H., Evangelista E. (1986). Dynamic recovery and strain hardening in the hot deformation of type 317 stainless steel. Mater. Sci. Eng..

[33] Zhao R.L., Liu Y., Tian B.-H. (2011). High temperature deformation behavior of pure copper. Heat Treat. Met..

